# The Role of Equilibrium between Free Radicals and Antioxidants in Depression and Bipolar Disorder

**DOI:** 10.3390/medicines9110057

**Published:** 2022-11-14

**Authors:** Anastasia Kotzaeroglou, Ioannis Tsamesidis

**Affiliations:** 1 Department of Biomedical Sciences, Metropolitan College, Campus of Thessaloniki, 54624 Thessaloniki, Greece; 2 School of Dentistry, Faculty of Health Sciences, Aristotle University of Thessaloniki, 54124 Thessaloniki, Greece

**Keywords:** free radicals, oxidative stress, depression, bipolar disorder, antioxidants, biomarkers

## Abstract

**Background:** Increasing evidence suggests that the presence of oxidative stress and disorders of the antioxidant defense system are involved in a wide range of neuropsychiatric disorders, such as bipolar disorder, schizophrenia and major depression, but the exact mechanism remains unknown. This review focuses on a better appreciation of the contribution of oxidative stress to depression and bipolar disorder. **Methods:** This review was conducted by extracting information from other research and review studies, as well as other meta-analyses, using two search engines, PubMed and Google Scholar. **Results:** As far as depression is concerned, there is agreement among researchers on the association between oxidative stress and antioxidants. In bipolar disorder, however, most of them observe strong lipid peroxidation in patients, while regarding antioxidant levels, opinions are divided. Nevertheless, in recent years, it seems that on depression, there are mainly meta-analyses and reviews, rather than research studies, unlike on bipolar disorder. **Conclusions:** Undoubtedly, this review shows that there is an association among oxidative stress, free radicals and antioxidants in both mental disorders, but further research should be performed on the exact role of oxidative stress in the pathophysiology of these diseases.

## 1. General Introduction

In the various fields of biology and medicine, free radicals are known as reactive oxygen species (ROS) or reactive nitrogen species (RNS). Free radicals are molecules or molecular fragments containing one or more unpaired electrons, the presence of which usually makes them highly reactive [1]. The beneficial effects of ROS tend to occur at low to moderate concentrations and are physiologically involved in various cell signaling pathways. Oxidative stress (OS) results from an imbalance between ROS formation and an organism’s reduced ability to detoxify free radicals or repair the damage they cause [2]. It is known that ROS and oxidative stress may play a role in many neuropsychiatric disorders [3,4]. 

Mood swings during a person’s lifetime are common due to both stressful and pleasant events. However, when these fluctuations become more intense and severe and lead to psychological distress, it may be a symptom of an underlying affective disorder. The latter are distributed along a spectrum from unipolar depressive disorders to type I and type II bipolar disorders [5].

Depression, as the most common cause of disability, affects almost 16% of the world’s population. Its main symptoms include decreased mood and anhedonia, i.e., decreased capacity for pleasure. Although much attention has been paid to this multifactorial and heterogeneous disorder, its pathophysiology remains poorly understood [6]. Similar findings exist for bipolar disorder, a disorder characterized by both depressive and manic episodes. Bipolar disorder is a relapsing disorder that affects more than 1% of the world’s population and usually occurs at a young age; in fact, it is the leading cause of disability among young people [7].

Despite decades of efforts, psychiatry still lacks reliable biological markers to distinguish between the two disorders, whose phenomenology can be extremely similar [8]. Therefore, their association with oxidative stress would likely help to better understand the pathophysiology of these disorders and lead to new therapeutic approaches.

### 1.1. Oxidative Stress

Oxidative stress (OS) is a physiological phenomenon that occurs in the organism. Normal intracellular levels of ROS are maintained at low levels by various enzyme systems involved in in vivo redox homeostasis. For this reason, oxidative stress can be considered as an imbalance between pro-oxidants and antioxidants in the body [9]. The first use of the term seems to date back to 1956 in rubber chemistry. In 1970, to describe H_2_O_2_ addition to erythrocytes, the phrase “cells were subjected to oxidative stress” was used, and since 1985, the term oxidative stress has been used to denote oxidative damage to cells and organs [10].

#### 1.1.1. Free Radicals

A free radical is any atom or molecule that contains one or more single electrons in its outer layer [11]. Free radicals are physiological products of many metabolic pathways. Some are maintained in a controlled form because they participate in essential functions of the organism; on the other hand, there are free radicals in free form, which interact with various tissue components [12]. Free radicals, therefore, play a dual role in living organisms, i.e., they either participate physiologically in the functions of the organism or they affect and interact with tissues. The term “radical” was first introduced by Guyton de Morveau in 1976 [13]. Due to excess of free radicals, cells are destroyed. This is accomplished via cell membrane degradation and mutations in DNA, but they can also damage membrane proteins [14].

Pro-oxidants/oxidants are called ROS/RNS. The most important free radicals produced via metabolic pathways are oxygen-containing radicals (ROS). Both ROS and RNS can be classified into two groups of compounds, namely, radicals and non-radicals (H_2_O_2_, ONOO^−^). The oxygen molecule itself is a radical. Some examples of free radicals are peroxide (O_2_^−^), hydroxyl (OH^−^), alkoxyl radical (RO^−^), peroxyl (ROO^−^), peroxide and nitrogen monoxide (NO^−^, NO_2_^−^) [15]. The first four belong to ROS, while the last two belong to RNS. In addition to these two categories of free radicals, there are also the reactive chlorine/bromine species (Cl^−^ and Br^−^), as well as the reactive forms of sulfur, more specifically the sulfur radical (RS) [10].

#### 1.1.2. Antioxidants

In contrast to redox homeostasis, the body’s defense against harmful levels of oxidants/free radicals is based on the mechanism of the antioxidant network. The latter consists of various antioxidant enzymes and low-molecular-mass antioxidants [16]. They have a vital role because of their ability to neutralize or destroy ROS or free radicals before they have a chance to destroy cells [14].

Antioxidants are divided into five main categories. Carotenoids are fat-soluble pigments responsible for the color of a wide variety of foods and can be classified into two broad categories, xanthophylls, oxygen-containing molecules such as zeaxanthin and lutein, and carotenes, non-oxygenated molecules such as alpha-carotene and lycopene [17]. Another category is flavonoids, which are divided into anthocyanins, flavonols, isoflavones and flavones. Flavonoids are a very important class of natural products and are essential ingredients in many nutritional and pharmaceutical applications due to their antioxidant, anti-inflammatory, anti-mutagenic and anti-cancer properties [18]. The third category is vitamins, more specifically A, C and E [19]. Finally, a separate group is resveratrol and tannins, containing ellagic acid, a potent antimicrobial compound [20,21].

#### 1.1.3. Classification of Oxidative Stress

Although Helmut Sies first attempted to classify OS in the past, in the 1980s, 40 years later, scientists have still not come up with an acceptable classification [22]. Because of the great variety and range of pro-oxidants, antioxidant enzymes and compounds, further attempts have been made to classify the sub-forms of oxidative stress and to introduce conceptual intensity scales ranging from normal to excessive and toxic oxidative stress [23]. According to Lushchak, OS can be classified based on its intensity into four subcategories. Basic OS (BOS), low-intensity OS (LOS), intermediate-intensity OS (IOS) and high-intensity OS (HOS). On the other hand, he proposes another categorization which includes mild (MOS), temperate (TOS) and severe (strong; SOS) oxidative stress.

#### 1.1.4. Factors Affecting Oxidative Stress

There are undoubtedly many factors that cause an increase in OS. Due to various diseases, patients experience increased levels of oxidative stress. One such disease is cancer, especially breast cancer, prostate cancer, lung cancer and various leukemias such as acute myelogenous leukemia, as well as Hodgkin’s and non-Hodgkin’s lymphomas [15,24]. Other diseases that cause an increase in OS are neurodegenerative diseases such as Alzheimer’s, Parkinson’s and amyotrophic lateral sclerosis [25,26]. In addition, acute exposure to solar UV radiation affects biomarkers related to oxidative stress in the skin, liver and blood [27]. Over time, it has been found that stress is associated with oxidative stress and that stress is also associated with a number of psychiatric disorders, such as depression, panic attacks, obsessive–compulsive disorder and post-traumatic stress disorder [28].

### 1.2. Depression

According to the WHO (World Health Organization), depression is a common disease affecting about 280 million people worldwide. During depressive episodes, patients experience sadness and irritation; they lose interest easily; and the things that used to make them happy no longer give them the same pleasure [29]. Other symptoms may include difficulty in concentrating, guiltiness, low self-esteem and hopelessness about the future, as well as sleep and weight disturbances. Finally, depression, in the worst cases, can lead to suicide and is the 4th leading cause of death for those aged between 15 and 29 years [30]. The diagnosis of depression is quite subjective, but experts rely on the Diagnostic and Statistical Manual of Mental Disorders (DSM-V) [31]. The most common form of depression is clinical depression or major depressive disorder (MDD), which is discussed in detail in the next section. Dysthymia is a form of chronic depression, also called persistent depressive disorder. Specifically, it is a relapsing depressive disorder with no clear episodes. Its onset is insidious and can be diagnosed either in adolescence or in adulthood, although for a long time period it remains unrecognized and undiagnosed [32]. On the other hand, two opposite subtypes of depression are melancholic and atypical depression, where both are associated with changes in the activity of the hypothalamic–pituitary–adrenal axis (HPA). In the first case, there is an increase in cortisol, while on the other hand, atypical depression is characterized by a decrease in cortisol [33]. Furthermore, postpartum depression is a type of depression that affects women, and it is one of the most common complications of childbearing [34]. Finally, seasonal depression is characterized by depressive episodes mainly in the autumn and winter months [35]. Major depressive disorder is a common psychiatric illness with high levels of morbidity and mortality. Among the general population, it is estimated that between 10% and 15% develop clinical depression in their lifetime. Studies conducted in different types of families (twins or adopted children) have shown that genetic factors play an important role in the development of MDD [36]. Moreover, environmental factors contribute to the developing of MDD, such as sexual, physical or emotional abuse during childhood [37,38]. Additional evidence from previous decades suggests that a neurotransmitter, serotonin, is implicated in this disease and that a dysfunction of serotonin receptors may play a role in the genesis of this disease; hence, many drug treatments rely on this mechanism [39]. Several studies have been conducted to enable scientists to come up with the most effective treatment. One treatment approach involves medication, such as antidepressants. The first line of therapy includes selective serotonin reuptake inhibitors (SSRIs) and serotonin–norepinephrine reuptake inhibitors (SNRIs). A second line of therapy due to side effects is the use of tricyclics, while as a third line of treatment, there are drugs with noradrenergic/dopaminergic action, such as mirtazapine or bupropion. However, patients do not show sufficient response to treatment, so there is a need for other therapeutic approaches [40]. The 12-month prevalence of major depressive disorder, although varying considerably from country to country, is around 6% overall. Evidence suggests that one in five people experience a depressive episode in their lifetime, and one in ten experience various symptoms of depression. Furthermore, it appears that modern lifestyles or poverty do not play any particular role in the prevalence of MDD, as high-income countries have a prevalence of 5.5%, while other low- or middle-income countries have a prevalence of 5.9%. Finally, there seem to be differences in rates between genders as well, with women showing higher prevalence rates, almost twice as high as men’s, with a possible justification being the difference in emotions and sensitivity between the two genders [41].

### 1.3. Bipolar Disorder

Bipolar disorder (BP) is a severe mental disorder that involves certain recurrent episodes, either characterized by increased or decreased mood, accompanied by changes in the activity or energy of people with this illness [42]. Episodes characterized by an elevated mood are called manic or hypomanic episodes, and depressive episodes are those where a decreased mood is detected, while euthymia is characterized by a balance in moods [43]. Bipolar disorder appears to carry a high degree of disability and has the highest suicide rate of all other psychiatric conditions, 20 to 30 times that of the general population [44]. The term bipolar was first used in 1957 by Leonard for disorders containing manic and depressive episodes. However, in 1980, the term bipolar disorder was formally adopted to replace the term manic depression in the DSM [45]. The risk factors for developing bipolar disorder are numerous and fall into two broad categories, environmental factors and genetic factors. Child abuse belongs to the first category, and many studies have shown that children who have grown up in an abusive environment and have been physically, sexually and emotionally abused can develop bipolar disorder or other mental illnesses [46,47,48]. Another environmental and genetic factor is substance abuse, such as cannabis, cocaine, opioids, alcohol and sedatives, although the exact mechanism remains unknown [49,50,51]. The DSM currently includes four main categories of bipolar disorder. There is BP type I, which is characterized by episodes of depression and at least one episode of mania, BP type II, which is also characterized by episodes of depression and at least one episode of hypomania but not mania. The last two types are cyclothymic disorder and not-otherwise-specified bipolar disorder (NOS) [45]. Cyclothymia appears to be characterized by the chronic presence of hypomanic episodes and low-level depressive symptoms [52]. In the scientific community, the best treatment for bipolar disorder is the combination of psychopharmacology and psychotherapy. The most effective drug for the treatment of manic episodes appears to be lithium carbonate. However, in addition to this, certain anticonvulsants, such as sodium valproate, carbamazepine and lamotrigine, are also used for treatment; the latter in particular helps in prevention. The mechanism by which anticonvulsants work is not yet known, although they sometimes have better results than lithium [53]. Also particularly useful for mood stabilization are atypical antipsychotics such as olanzapine, risperidone and quetiapine, but unfortunately, there are insufficient data to distinguish atypical antipsychotics, mainly due to heterogeneity [54]. Epidemiological studies seem to have shown that the prevalence of BP-I is 1% in the general population. One study that included 11 countries found the prevalence to be 2.4% with 0.6% for BP-I and 0.4% for BP-II [55]. In the USA, the prevalence was found to be 1%, slightly higher than the other countries. This may be either because more stringent diagnostic measures are used in America or there is indeed an increased number of people with bipolar disorder. In addition, a meta-analysis that was mainly performed in America found prevalence rates of 1.06% and 1.57% for BP-I and BP-II, respectively. However, similar findings were found in studies conducted in Germany and Italy [56]. BP-I appears to have equal prevalence in both men and women [57]. However, some studies suggest that the rate is higher in women than in men [58], while other research states that bipolar disorder affects men more than women [59].

## 2. Methodology

A comprehensive literature search was conducted from October 2021 till July 2022 using PubMed, Scopus and Google Scholar. The literature review was conducted using the following pre-specified search terms: depression AND oxidative stress, bipolar disorder AND oxidative stress, antioxidants AND depression, antioxidants AND bipolar disorder, reactive oxygen species AND depression, reactive oxygen species AND bipolar disorder. Two reviewers independently searched the literature, applied screening filters, and evaluated each article’s eligibility for inclusion based on the predefined criteria. Two additional reviewers supervised the study selection process.

## 3. Oxidative Stress in the Mental Disorders under Study

### 3.1. The Role of Free Radicals in Major Depressive Disorder

**Figure 1:**
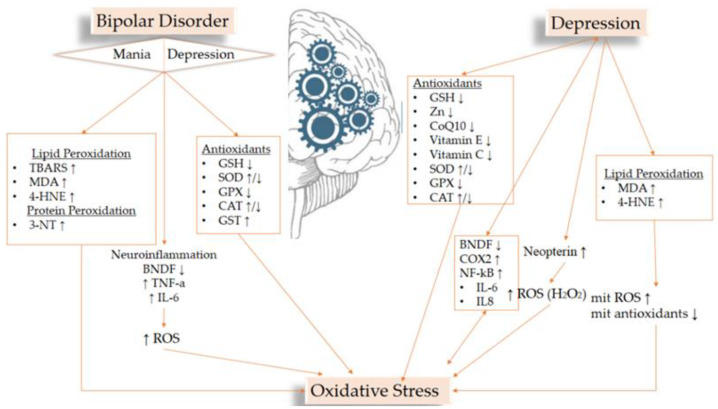
Flowchart of the most important results related to OS biomarkers and the studied disorders.

Many papers report that oxidative stress is inextricably linked to mental disorders [60,61,62]. It is argued that due to the intense activity of the brain and thus its need for large amounts of oxygen, due to its high lipid content and because of its low antioxidant defense, it is particularly vulnerable to oxidative stress. Oxidative stress plays a significant role in neurodegeneration and is thus associated with many neurodegenerative and neuropsychiatric diseases [63]. The flowchart (Figure 1) summarizes the results below.

Table 1 summarize the OS biomarkers used in various studies studying depression. The involvement of oxidative stress in major depressive disorder is undoubtedly documented; more specifically, OS and proinflammatory signaling have emerged as key axes in the pathogenesis of MDD, which are also interconnected [63]. Studies have shown that increased ROS production and reduced antioxidant defenses lead to brain alteration in people with MDD, such as reductions in the frontal cortex and the hippocampus [64]. In addition, OS destroys neuronal DNA in the brain; therefore, it causes DNA strand fragmentation and sister chromatid exchange [65]. Increased ROS also upregulate the hypothalamic–pituitary–adrenal axis (HPA) and alter the transmission of γ-aminobutyric acid and serotonin [28].

Oxidative stress leads to the activation of pro-inflammatory pathways. MDD is associated with an imbalance between several factors, such as brain-derived neurotrophic factor (BDNF) and nuclear factor NF-kB [66]. Pro-inflammatory cytokines, such as interleukins 6 and 8, are activated by NF-kB. The activity of this transcription factor is regulated by the levels of ROS and glutamic acid. On the other hand, the transcription factor itself increases the levels of oxidative stress, causing an inflammatory response. Increased levels of NF-kB have been observed in patients with MDD, as shown in Table 1 [67].

Changes in inflammation markers appear in relation to the number of depressive episodes. In particular, neopterin is significantly elevated in patients with depressive episodes, which enhances the effect of ROS such as hydrogen peroxide (H_2_O_2_) [68]. 

Prostaglandin-endoperoxide synthase 2, also known as cyclooxygenase-2 or COX-2, represents an enzyme involved in inflammatory processes in the body. COX-2 inhibitors cause the inflammation of neurons, which can lead to the worsening of depression. In addition, they can damage mitochondria and lead to lipid peroxidation and the reduction in antioxidants [67]. COX-2 production inhibitors can exacerbate five pathways: they may cause neuroinflammation, cause Th1 translocation, increase OS, decrease antioxidant defense and impair mit functions [69].

Lipid peroxidation is the result of increased damage caused by OS. Therefore, due to this, there is an increase in the levels of malondialdehyde (MDA) (Table 1), which is a by-product of polyunsaturated fatty acid (PUFA) peroxidation, and of arachidonic acid and increased expression of 4-hydroxynonenal (4-HNE) [70]. MDA inhibits the nucleotide repair system resulting in DNA becoming more susceptible to mutations, making it easier to destroy. In addition, these increased levels also cause damage to mitochondrial DNA, increasing the levels of mitochondrial ROS (ROS produced by the mit ETC chain, as there are multiple sources of ROS in the cell) while decreasing mitochondrial antioxidant levels [71,72]. Therefore, oxidative stress can damage fatty acids, which in turn cause lipid peroxidation and damage to brain cell membranes.

### 3.2. Levels of Oxidative Stress in Bipolar Disorder

On the other hand, in bipolar disorder, what has been mainly observed is increased lipid peroxidation (MDA) summarized in Table 2. Mitochondria are intracellular organelles responsible for the production of ATP through oxidative phosphorylation via the electron transport chain. In BD, certain changes in these pathways appear to lead to an increase in ROS, which causes damage to lipids, DNA and proteins [73]. Post mortem studies using the brains of BD patients have shown reduced expression of mitochondrial electron transport chain (ETC) genes. Mitochondrial ROS production has been shown to lead to telomere shortening [74], and this phenomenon has been observed in humans with bipolar disorder [75]. Thus, Andreazza and her colleagues have suggested that the relationship among mit ETC (electron transport chain) dysfunction, OS, cell death and DNA damage is a promising area for investigating the pathophysiology of BD [76].

Researchers have understood that there is lipid damage in BD patients by increasing the levels of thiobarbituric acid reactive substances (TBARSs). TBARSs are lipid peroxidation products and can be considered a direct indicator of lipid damage [77]. TBARSs appear to be elevated in both mania and euthymia, and for this, they cannot be considered as a marker of BD status [78]. In comparison, TBARSs are more elevated in mania [79,80]. Increased lipid damage more specifically was found to be present in the prefrontal cortex and anterior cingulate cortex of BD patients [81].

The oxidation of proteins can also lead to devastating consequences through the formation of harmful intermolecular aggregates and the alteration of protein function. It has also been reported that carbonylation and the levels of 3-nitrotyrosine (3-NT), which is a biomarker of oxidative stress, were associated with decreases in ETC I complex activity [80]. These data suggest that oxidative damage to proteins is increased in BD due to an increase in 3-NT and that it is likely to be associated with mitochondrial dysfunction [82]. Research has shown that 3-NT is elevated mainly during the phase of depressive episodes [83], while other researchers have shown that there is no statistically significant difference [73]. In addition, it has been noted that 4-HNE is also elevated in BD patients [84]. 4-HNE is produced via the peroxidation of ω-6-polyunsaturated fatty acids and is one of the main aldehydes produced due to OS [85]. Finally, it has been reported that lipid peroxidation is more pronounced during mania [79]. 

Many studies have noted that nitric oxide (NO) is also highly elevated in individuals with BD [77,78,81]. They have shown that nitric oxide levels are found to be elevated in mitochondrial proteins in patients with BD [86]. NO is a paracrine factor that is derived from the endothelium and controls vascular tone. Increased ROS concentrations reduce the amount of bioactive NO [87]. 

Evidence suggests that the presence of OS may be associated with the overactivation of glutaminergic and dopaminergic systems in BD. Glutamate plays an important role in mediating oxidative balance. More specifically, glutaminergic overactivity leads to increased calcium flux, resulting in increased OS [69]. In addition, OS may increase due to dopamine excess because its metabolism leads to ROS production [88]. Finally, other systems that act with BD are serotonergic and γ-aminobutyric acid (GABA), which were found to have reduced activity. OS is associated with reduced GABA release, while increased serotonin metabolism, which reduces serotonergic function, was found to increase OS levels [89].

Serum BDNF has been found to be decreased in BD during manic or depressive episodes when comparing patients in the euthymia phase and healthy controls. The more episodes a patient has experienced, the lower BDNF levels they have [90]. Furthermore, neuroinflammation appears to cause dysfunction in many neurotransmitter and neuronal signaling systems, and proinflammatory cytokines such as TNF-a and IL-6 are stimulated during mania and activate microglia; these in turn release ROS, leading to oxidative damage, protein accumulation and apoptosis. These changes in neuroglial function reduce various factors, such as BDNF [91].

### 3.3. Antioxidant and MDD

#### 3.3.1. The Role of Antioxidants in Mental Disorders

Table 3 summarizes the studied antioxidant levels in MDD patients. MDD is accompanied by reduced concentrations of antioxidants, including coenzyme Q10 (CoQ10), vitamin E, zinc and glutathione (GSH) [63]. Reduced levels of the latter lead to the pathogenesis of depression, as it is one of the main free radical scavengers. It is a tripeptide composed of the amino acids cysteine, glycine and glutamine and has high antioxidant capacity [92,93].

In addition, CoQ10 is a powerful antioxidant that has anti-inflammatory activity and reduces pro-inflammatory cytokines; reduced levels have been observed in the plasma of people suffering from depression. This coenzyme, because of its antioxidant and anti-inflammatory properties, may be considered as an alternative drug for the treatment of depression [94,95]. Vitamin E is a fat-soluble antioxidant whose main property is to protect the brain from oxidative damage by removing free radicals. In MDD, in addition to low levels of vitamin E, low levels of vitamin C are also observed. The latter is a water-soluble antioxidant that, through the restoration of reduced vitamin E, is effective in reducing oxidative stress [96]. Finally, N-acetylcysteine (NAC) is an antioxidant that acts as an ROS scavenger and appears to be effective mainly for hydroxyl radicals. With the help of NAC, glutathione levels are increased, because it is a precursor molecule of glutathione and has the ability to cross the blood–brain barrier and replenishes GSH. Therefore, it seems that NAC protects the brain from oxidative stress markers, and studies have shown that it relieves the symptoms of depressed patients [97]. However, although cysteine is an amino acid that can be found in many foods, it is not naturally present in the body in the form of NAC [98], so it is used as a dietary supplement, which has been shown to be helpful in the symptoms of depression.

Regarding the function of antioxidant enzymes in depressed patients, it appears that superoxide dismutase (SOD) is observed to be increased or decreased in response to high superoxide concentration [63]. (Bhatt et al., 2020) More specifically, it appears that in red blood cells, SOD is elevated, while in serum and plasma, its levels are decreased [99]. According to Bhatt and his colleagues the same seems to be true for the enzyme catalase (CAT). SOD and CAT are antioxidant enzymes that are responsible for the elimination of free radicals, such as peroxide and hydrogen peroxide, and possess antioxidant mechanisms [100].

Another antioxidant enzyme is glutathione peroxidase (GPX), which was found to be reduced in depressed subjects compared with the healthy group [101]. Decreased activity causes ROS accumulation and is negatively correlated with the severity of depression and its symptoms. GPX protects the body from cell death but also protects DNA and neurons from various damages [67].

On the other hand, some studies do not agree with the above findings, claiming that there no particular decrease in GPX could be found; furthermore, regarding CAT levels, they found no particular difference in people with depression compared with the healthy group [102,103,104].

According to Gautam and his colleagues, it seems that combination therapy with antioxidants and antidepressant/anti-anxiety drugs has a particular effect on people with MDD [105]. Moreover, according to Bhatt and his colleagues, antidepressants affect, in addition to the concentration of neurotransmitters, the levels of oxidants and antioxidants. In addition, nutritional supplements such as vitamins, n-3 PUFA and antioxidants reduce but also slow down the symptoms and progression of MDD.

#### 3.3.2. The Contribution of Antioxidants to Bipolar Disorder

Table 4 summarizes the studied antioxidant levels in BD patients. ROS generated along the ETC are detoxified by antioxidant enzymes. Alterations in these enzymes have been reported in BD patients [69]. Some studies have found no particular difference in the levels of antioxidant enzymes such as SOD, CAT, GPX and glutathione S-transferase (GST) [74,77,86,106].

On the other hand, studies have reported a difference only in SOD, with it being increased, more specifically in the acute phases of BD (mania and depression) and not in euthymia [80,107,108], while other studies have found decreased concentrations of SOD [106,107]. However, Yumru and his colleagues have pointed out that SOD is increased in patients with mania and euthymia and decreased in patients with depression.

In addition, other researchers have shown that there is a difference in CAT, either decreased levels [106] or increased levels [78,89]. The latter even noted that there were both increased levels of GST and decreased levels of GPX in patients with mania, while increased levels of GPX were found by de Sousa and his colleagues. However, whether or not they found statistically significant differences in SOD and CAT, they suggested that the combination of the two may be used as a state marker [78].

In Section 3, it is mentioned that drugs affect BD; methamphetamine has been found to lead to ROS production and cause oxidative stress, affecting the antioxidant system of people [108]. Furthermore, research has reported that amphetamine is associated with an imbalance between SOD and CAT during mania in an animal model of rats [109].

As for GSH, its concentration seems to increase in bipolar patients according to Das and his colleagues [110], while other researchers have reported that in mania and euthymia, the levels decrease [78]. A decrease in its levels has also been observed by another study of the plasma of patients. These researchers argue that a decrease in the total level of GSH is not a direct biomarker of a patient’s psychotic state but rather indicates the predisposition they may have to a psychotic episode [111]. As already mentioned, glutamate plays an important role in oxidative balance, as it is one of the three amino acids that contribute to the production of GSH, so disturbances in the glutamate pathway affect its levels [69].

Moreover, as mentioned in the previous chapter, NAC is a potent antioxidant and a precursor molecule of glutathione and can replace the reduced GSH concentration [112]. Studies have shown that it is also quite effective in BD symptoms and especially in treating the depressive episodes of BD, for which finding a treatment is imperative, and NAC seems to help in the acute form of BD [113]. However, studies suggest some issues about NAC that remain unresolved and need full clarification [114].

Finally, studies have reported that the medication already used to treat BD appears to have antioxidant properties and may help to reduce OS, such as lithium, valproic acid, lamotrigine and quetiapine [69]. Research has shown that lithium and valproate increase GSH levels and thus enhance antioxidant defense [115]. Moreover, GSH plays an important role in the interaction among lithium, mitochondrial dysfunction and OS [116]. However, other studies have reported that lithium contributes to oxidative stress and even increases its levels [117,118].

Therefore, there is a discrepancy in the bibliography regarding antioxidant levels, which indicates the specificity of the oxidative status of BP patients and the need for further study.

## 4. Discussion

Undoubtedly, this review shows that there is an association among oxidative stress, free radicals and antioxidants in both depression and bipolar disorder. In the former disease, there is more clarity and agreement among researchers; for example, there is a decrease in antioxidants such as vitamin E, CoQ10, zinc and GSH, while regarding the antioxidant enzymes, there is disagreement on their levels. On the other hand, regarding BD, researchers seem to disagree on the levels of antioxidant enzymes, while most of them highlight the strong presence of TBARSs due to lipid peroxidation.

A major limitation is the great heterogeneity observed in the studies, such as the different protocols followed by each investigator and the different levels of OS between depressed and bipolar patients. Some studies seem to ignore the treatment each patient is following when studying oxidative balance. Another gap is that in depression, people with MDD have been studied, but there has not been much research on the other types of depression. On the other hand, in BD, things may be more confusing, since patients were in different phases when the reviewed studies were conducted, as a bipolar individual may be in the mania, depression or euthymia phase. It seems that the levels of antioxidant enzymes differ in each phase. In addition, researchers should note and take into account differences that may be observed due to the medication the patient is on when taking part in the studies.

Nevertheless, most studies agree that for both diseases, and especially in BD, further research into the pathophysiology is needed to adapt treatment approaches and discover new strategies that may be more effective than the existing ones, which seem to be inadequate. One promising treatment seems to be the use of antioxidants, especially for the treatment of depressive symptoms, for instance, NAC. The identification of distinct biomarkers is of particular importance as they would help to assess the severity, the prediction of changes, the course and the neurobiology of the diseases.

After our review, we found a high incidence rate of bipolar disorder, but many patients with BD are not aware of it. This may be due to either negligence of the symptoms by the patients themselves or due to misinterpretation by experts; as already mentioned, many patients are diagnosed with depression, MDD, and not BD. This is also a major limitation of this study, as it is not known whether the patients involved in the trials reported in this review were correctly diagnosed.

Future studies should measure OS biomarkers and antioxidants at the same time, but there is a need to measure them in different blood sources, such as red blood cells, serum and plasma. Furthermore, in studies where the efficacy of antioxidants in mental illnesses is examined, there is a need to select patients based on a certain predefined minimum oxidative status.

## 5. Conclusions

Overall, this review shows that there is a correlation among oxidative stress, antioxidant defense and the two psychiatric disorders. However, several questions still remain unanswered, and with respect to some biomarkers, there are discrepancies among studies in terms of their levels, so there is a need to conduct further research for better clarification. Ultimately, deepening our knowledge of the mechanism of oxidative stress in the brain would lead to the rational development of new therapeutic approaches and/or biomarkers for the treatment of psychiatric diseases.

## Figures and Tables

**Table 1:** Summary of OS biomarkers in depression.

Biomarker of OS	Number of Participants	Results	Observations	References
*MDA*	Review	Increase in MDA	By-product of PUFA peroxidation.	Yoshida et al. (2013)
*4-HNE*	Review	Increase in aldeyde levels (4-HNE)	Main aldehyde produced due to OS.	Yoshida et al. (2013)
*ROS*	Review	Increase in ROS	Alteration of the brain in people with MDD.	Belleau et al. (2019)
*RNS*	Review	Increase in RNS	-	Belleau et al. (2019)
*NFkB*	Review	Increase in ROS levels	Its activity is influenced by the levels of ROS and glutamic acid.	Vaváková et al. (2015)
*BDNF*	Review	Decrease in BDNF	-	Bakunina, et al. (2015)
*Neopterin*	Meta-analysis	Increase in Neopterin	Enhances the effect of ROS.	Moylan et al. (2013)
*COX-2*	Review	Increase in COX-2	COX-2 inhibitors lead to lipid peroxidation and reduction in antioxidants.	Vaváková et al. (2015)

Abbreviations used: MDA, malondialdehyde; 4-HNE, 4-Hydroxynonenal; ROS, reactive oxygen species; RNS, reactive nitrogen species; NFkB, nuclear factor kappa b; BDNF, brain-derived neurotrophic factor; COX-2, cyclooxygenase 2; PUFAs, polyunsaturated fatty acids; OS, oxidative stress; MDD, major depressive disorder.

**Table 2:** Summary of OS biomarkers in bipolar disorder.

Biomarker of OS	Number of Participants	Results	Observations	References
*MDA*	Meta-analysis	Increase in MDA	-	Brown et al. (2014)
*TBARS*	212	Increase in TBARSs	In the phase of mania and euthymia.	Kunz et al. (2008)
*3-ΝΤ*	155	Increase in 3-NT	During depressive episodes.	Kapczinski et al. (2011)
*4-HNE*	60	Increase in 4-HNE	-	Wang et al. (2009)
*NO*	60	Increase in NO	Nitrosylation levels were found to be increased in mitochondrial proteins of patients with BD.	Andreazza et al. (2013)
*BDNF*	Review	Decrease in BDNF	During depressive and manic episodes.	Rowland et al. (2018)

Abbreviations used: MDA, malondialdehyde; TBARSs, Thiobarbituric acid reactive substances; 3-ΝΤ, 3-nitrotyrosine; 4-HNE, 4-Hydroxynonenal; NO, *Nitric oxide*; BDNF, brain-derived neurotrophic factor; OS, oxidative stress; BD, bipolar disease.

**Table 3:** Summary of antioxidant levels in MDD.

Antioxidants	Number of Participants	Results	Observations	References
*CoQ*	45	Decrease	Alternative medicine for treating depression.	Sanoobar et al. (2015)
*Vitamin E*	41	Decrease	Protects the brain from oxidative damage.	Mazloom et al. (2013)
*Vitamin C*	41	Decrease	Effective in reducing OS.	Mazloom et al. (2013)
*Zn*	Review	Decrease	-	Bhatt, et al. (2020)
*GSH*	Review	Decrease	Decreased levels can lead to the pathogenesis of depression.	Bhatt, et al. (2020)
*SOD*	100	Increase/Decrease	It is increased in red blood cells, while serum and plasma levels are decreased.	Camkurt et al. (2016)
*GPX*	51	Decrease	Reduced activity of GPX causes ROS to accumulate.	Stefanescu and Ciobica, (2012)
*Catalase*	Review	Increase/Decrease	-	Bhatt, et al. (2020)

Abbreviations used: CoQ10, coenzyme q10; Zn, Zinc; GSH, glutathione; SOD, sodium oxide dismutase; GPX, glutathione peroxidase; OS, oxidative stress; ROS, reactive oxygen species; MDD, major depressive disorder.

**Table 4:** Summary of antioxidant levels in BD.

Antioxidant	Number of Participants	Results	Observations	References
*GSH*	114	Decrease in GSH	Indicates the predisposition a patient may have to a psychotic episode.	Nucifora et al. (2017)
*SOD*	-	-	Discrepancy among studies.	-
*GPX*	-	-	Discrepancy among studies.	-
*Catalase*	-	-	Discrepancy among studies.	-
*GST*	Meta-analysis	Increase in GST	-	Jiménez-Fernández et al. (2020)

Abbreviations used: GSH, glutathione; SOD, sodium oxide dismutase; GPX, glutathione peroxidase; GST, glutathione S transferase; BD, bipolar disease.

## Data Availability

Data are contained within the article.
